# Body mass index affects EEG microstate dynamics through blood viscosity in high-altitude environments

**DOI:** 10.3389/fnins.2026.1829857

**Published:** 2026-06-19

**Authors:** Zian Liu, Yongjun Jing, Runqing Lu, Keqi Song, Niannian Wang, Hao Li

**Affiliations:** 1 Xizang Autonomous Region Key Laboratory for High Altitude Brain Science and Environmental Acclimatization, Xizang University, Lhasa, China; 2 China Railway No. 10 Engineering Group Co., Ltd., Lhasa, China

**Keywords:** blood viscosity, body mass index, EEG microstates, high altitude, hypoxia, microstate B, resting-state EEG

## Abstract

High-altitude hypoxia imposes substantial challenges on cerebral oxygen delivery and brain functional regulation. Body mass index (BMI) may influence neurophysiological adaptation to such environments through metabolic and hemorheological pathways, yet its relationship with resting-state Electroencephalography (EEG) microstate dynamics remains unclear. This study examined whether blood viscosity (BV) mediates the association between BMI and EEG microstate characteristics in a high-altitude population. A total of 123 permanent residents from Lhasa, Tibet, were initially included. Because only five participants met the obesity criterion, the main statistical analyses were conducted in 118 participants after excluding the obese subgroup. Resting-state eyes-closed EEG data were recorded using a 64-channel system, and standardized 180-s artifact-free segments were used for microstate analysis. Four canonical microstates were identified, and temporal parameters, including coverage, duration, global explained variance, global field power, occurrence, and transition probabilities, were extracted. BMI was calculated from measured height and weight, and BV was estimated using hematological indicators based on a validated high-altitude prediction model. The four identified microstate maps showed topographic patterns broadly consistent with previous EEG microstate studies. Microstate C showed the highest values across several temporal parameters, suggesting a potentially dominant resting-state pattern under high-altitude conditions. BMI was positively correlated with BV, while BV was negatively correlated with the occurrence of microstate B. The mediation model suggested an indirect statistical pathway from BMI to microstate B occurrence through estimated BV: higher BMI was associated with higher estimated BV, which in turn was associated with reduced microstate B occurrence. These findings suggest that BMI-related alterations in resting-state brain dynamics at high altitude may be linked to hemorheological changes. Overall, this study provides preliminary evidence for a BMI–blood viscosity–brain microstate pathway in high-altitude residents. The results highlight BV as a potential physiological bridge between body composition and spontaneous brain activity under chronic hypoxic exposure.

## Introduction

High-altitude environments are characterized by hypoxia, low temperatures, and strong ultraviolet radiation, which pose significant challenges to physiological homeostasis. Body mass index (BMI), as a core indicator for assessing obesity, undergoes adaptive changes in high-altitude environments due to hypoxia-induced metabolic reprogramming (such as abnormal fat distribution and reduced muscle mass) (Díaz-Gutiérrez et al., 2016; Moore, 2017), but its long-term impact on neurocognitive function remains unclear. Studies have shown that increased BMI among high-altitude residents is associated not only with brain structural abnormalities such as hippocampal atrophy and prefrontal cortical thinning (Koenig and Brandeis, 2016), but also with dysregulation of cerebrovascular hemodynamics (Yao et al., 2017), suggesting that BMI may influence brain functional networks through multisystem interactions. In addition, evidence from normoxic and low-altitude settings suggests that obesity or elevated BMI is related to abnormal hemorheology and altered cerebral perfusion, indicating that BMI may affect brain function not only through metabolic and inflammatory pathways, but also through vascular and oxygen-delivery mechanisms (Guiraudou et al., 2013; Qiao et al., 2022). These observations are particularly relevant in high-altitude environments, where hypoxia places greater demands on cerebral blood flow regulation and oxygen transport efficiency (Liu et al., 2017).

Even in the absence of explicit tasks, the human brain exhibits complex spontaneous activity patterns, a phenomenon known as resting-state functional connectivity (FC). These patterns reflect the brain’s intrinsic functional structure, where coherent fluctuations in neural activity across different brain regions form identifiable networks (Glasser et al., 2016). Scientific research has revealed striking correspondences between resting-state FC and task-related brain activity, indicating the existence of an intrinsic functional structure underlying most brain activity (Guerra-Carrillo et al., 2014). Brain microstates, as transient neural activity patterns derived from resting-state Electroencephalography (EEG) analysis, reflect the dynamic reconfiguration capacity of brain functional networks (Lehmann et al., 1987). Among them, microstate B has commonly been linked to visual-network-related processing and visual imagery, although its precise functional interpretation may vary slightly across studies and analytic pipelines (Michel and Koenig, 2018; Tarailis et al., 2024). Because visual information processing and attention are among the cognitive domains that are vulnerable to obesity-related neural inefficiency, microstate B may be a relevant electrophysiological marker when examining BMI-related alterations in spontaneous brain activity. Recent resting-state EEG evidence further suggests that overweight and obesity are associated with altered microstate dynamics, including reduced expression of microstate B, providing a more direct rationale for examining the association between BMI and microstate B in the present study (Mi et al., 2026). Abnormalities in its spatiotemporal characteristics are significantly associated with cognitive fatigue, neurodegenerative diseases, and psychiatric disorders (Chu et al., 2020; Yee et al., 2021). However, how high-altitude hypoxia regulates microstate dynamics through BMI has not been studied. Existing evidence suggests that hypoxic exposure can disrupt microstate sequences and functional connectivity among brain regions (Zhang et al., 2023), but the mechanistic role of BMI in this process remains to be elucidated.

As a key parameter of hemorheology, blood viscosity (BV) is significantly elevated in high-altitude hypoxic environments due to compensatory increases in red blood cells and decreased plasma volume (Huamaní et al., 2022). Elevated blood viscosity not only increases the risk of cardiovascular disease (Jiang et al., 2018; Mach et al., 2020), but also directly interferes with neuronal synchronized activity by reducing cerebral blood flow velocity and oxygen delivery efficiency (De Simone et al., 1990; Marioni et al., 2010). Notably, studies under normal low-altitude conditions have shown that obesity is associated with increased whole-blood viscosity, plasma viscosity, and erythrocyte aggregation, which provides an important physiological basis for proposing a BMI–blood viscosity–brain function pathway (Guiraudou et al., 2013). Moreover, systematic evidence suggests that obesity is associated with alterations in cerebral blood flow (Qiao et al., 2022), while resting-state neuroimaging studies indicate disrupted intrinsic functional organization in obesity (Parsons et al., 2022). Although direct studies simultaneously examining BMI, blood viscosity, and EEG microstates under normoxic conditions remain scarce, the available evidence supports the plausibility of such a pathway. Notably, the increase in blood viscosity is more pronounced in obese individuals, and the dynamics of their brain states show a mediating association with cognitive impairment (Han et al., 2024), suggesting that BMI may indirectly modulate neural activity patterns via blood viscosity. However, this hypothesis has not yet been validated in high-altitude environments.

Therefore, this study focuses on the uniqueness of high-altitude hypoxic environments and proposes the following scientific hypotheses: (1) We hypothesized that higher BMI would be associated with lower microstate B occurrence, based on the functional relevance of microstate B to visual-network-related processing and emerging evidence that overweight/obesity is accompanied by reduced expression of microstate B (Michel and Koenig, 2018; Mi et al., 2026). (2) Blood viscosity was expected to statistically mediate the relationship between BMI and microstate B, that is, BMI increases blood viscosity, indirectly leading to abnormalities in the distribution of microstate B; this hypothesis is grounded in prior low-altitude findings showing that elevated BMI is associated with abnormal hemorheology and altered cerebral perfusion (Guiraudou et al., 2013; Qiao et al., 2022). By integrating BMI, estimated blood viscosity, and EEG microstate data, this study provides a preliminary observational framework for examining a potential BMI–blood viscosity–brain microstate pathway in high-altitude environments.

## Materials and methods

### Participants

This study randomly recruited 203 participants from Chengguan District, Lhasa, Tibet. EEG data and physiological indicators were collected from all 203 participants. Inclusion criteria included: not using medications related to the experiment, no undergoing surgeries related to experimental variables, right-handedness, good physical health, and no neurological or psychiatric disorders, brain injury, or substance addiction problems. Data from 80 participants were excluded because of incomplete physiological measurements, unusable EEG recordings, or missing key variables. After applying the above criteria, the final sample consisted of 123 participants. The average BMI of these participants (male: 19; female: 104) was 21.595 ± 3.168 (range: 16.41–31.20). This study was approved by the Ethics Committee of Tibet University. All participants provided written informed consent and received compensation for their participation.

### Measures and procedures

#### Baseline survey

(1) Physical examination: Height and weight were measured by uniformly trained personnel following a standardized protocol, and BMI was calculated as BMI = weight (kg)/[height (m)]^2^. According to the Guidelines for the Prevention and Control of Overweight and Obesity in Chinese Adults, BMI < 18.5 is underweight, 18.5–23.9 is normal, 24.0–27.9 is overweight, and ≥ 28.0 is obese (National Health Commission of the People’s Republic of China, 2021).

(2) Laboratory testing: Data were collected and recorded by professional physicians and advanced instruments at Tibet Fukang Physical Examination Center. Serum samples were drawn after overnight fasting for complete blood count measurements. Routine blood indices included fasting plasma glucose (FPG), hematocrit (HCT), globulin, and triglycerides (TG). Because direct blood viscosity measurement was not available in the present study, BV was estimated using a previously published high-altitude prediction model. BV was not directly measured in the present study but was estimated using a previously published high-altitude prediction model developed by Huamaní et al. (2022). The model was established in clinically healthy adults living in Cusco, Peru, a high-altitude city located at 3,399 m, and BV in the original study was measured using a Brookfield AMETEK cone–plate viscometer under controlled temperature conditions with daily calibration using a standard 5 cP viscosity solution. The simplified predictive equation included hematocrit, globulin, and triglyceride levels and explained 68.07% of the variance in BV. Therefore, the model was considered appropriate for estimating BV in the present high-altitude population. Estimated BV was calculated as follows:


Bloodviscosity(cP)=Hematocrit(%)×0.176+

$$\mathrm{Globulin}\,\left(\frac{d}{\mathrm{lg}}\right)\times 0.595+\mathrm{Triglycerides}\,\left(\frac{\mathrm{mg}}{\mathrm{dl}}\right)\times \left(\frac{1.772}{1000}\right)-4.129$$

Participants were then dichotomized into high- and low-BV groups based on the sample mean.

(3) EEG data acquisition and preprocessing.

EEG signals were acquired using the ANT Neuro 64-channel system (ANT Neuro B.V., Enschede, Netherlands) at a sampling rate of 1,000 Hz. Electrode placement strictly followed the international 10–20 system, and electrode impedances were kept below 5 kΩ. During online recording, CPz was used as the reference electrode and FCz as the ground electrode. Signals were continuously collected after amplifier gain adjustment. Online filtering parameters were set to a 0.01–100 Hz bandpass range. Participants underwent 5 min of data acquisition in an eyes-closed resting state.

Raw data processing was conducted on the MATLAB 2021b platform (MathWorks, Natick, MA), jointly using the EEGLAB (Delorme and Makeig, 2004) and FieldTrip (Oostenveld et al., 2011) toolboxes. The preprocessing pipeline included: first downsampling the data to 256 Hz to optimize computational efficiency, then applying a bidirectional finite impulse response (FIR) filter with a 2 Hz high-pass and 20 Hz low-pass. Abnormal electrode signals were corrected using a three-dimensional spherical interpolation algorithm, after which the continuous EEG was segmented into 500 ms time windows for manual artifact detection and rejection.

An improved independent component analysis (ICA) algorithm (Jung et al., 2001) was used for biopotential artifact correction. Specifically, based on the ICLabel classifier (Pion-Tonachini et al., 2019) with a probability threshold of 0.7, components related to electromyographic activity, electrocardiographic activity, powerline interference, and channel noise were systematically removed (an average of 4.8 ± 1.9 components removed) (Bagdasarov et al., 2022). After excluding abnormal segments using a ± 100 μV peak-to-peak threshold, the clean data segments were concatenated. To ensure comparability across participants, a fixed-length segment of artifact-free EEG data was selected for each individual. Specifically, approximately 3 min of continuous clean data (180 s) were extracted from the preprocessed recordings. This procedure ensured that all participants contributed equal-length EEG data to the subsequent analyses, thereby eliminating potential biases introduced by differences in data length across individuals or groups (e.g., high versus low blood viscosity groups). The final data were then converted to average reference and entered into the microstate analysis module.

(4) Microstate analysis.

Resting-state EEG data were analyzed using the Microstate Toolbox (Poulsen et al., 2018). First, the preprocessed EEG data were band-pass filtered at 2–20 Hz to remove low-frequency noise (such as eye movements and blinks) and high-frequency noise (such as muscle activity) (Kasim and Tosun, 2022; Seitzman et al., 2017). All microstate analyses were conducted on the standardized 180-s EEG segments for each participant, ensuring that microstate parameters were derived from data of identical duration across individuals. Subsequently, for each participant in each group, global field power (GFP) was computed, which is the standard deviation of EEG signals across all electrodes at each time point, representing the instantaneous strength of scalp EEG activity (Skrandies, 1990). Since EEG scalp topographies remain stable near GFP peaks, only the EEG topographies corresponding to the GFP peaks were extracted and submitted to the clustering algorithm. We used an improved k-means clustering algorithm to obtain topographic maps based on topographic similarity, while ignoring polarity. We applied this improved k-means clustering algorithm multiple times with the number of clusters k ranging from 2 to 8, thereby obtaining 2–8 topographic maps for each participant and each group. Next, a second round of topographic clustering was performed at the group level using individual topographic maps from different participants. Similarly, polarity was ignored in this process. The optimal number of clusters (four in this study) was determined by cross-validation, and the two sets of topographic maps were clustered into four microstates, respectively. Finally, the four microstate categories obtained at the group level were used as template maps, and based on the spatial correlation between the template maps and each participant’s scalp potential topography at each time point, each time point of each participant’s scalp potential topography was back-fitted into one of the four categories (A–D).

After obtaining participants’ microstates, three commonly used EEG microstate features—duration, occurrence, and coverage—were extracted from the microstate time series for subsequent analysis. Duration refers to the average length of time a given microstate category is detected in the EEG data, reflecting the stability of the potential neural configuration. Occurrence refers to the frequency with which a given microstate maintains dominance (number of presentations per second), representing the representational tendency of potential neural activation; coverage refers to the ratio of the total time a given microstate maintains dominance to the total recording time. Finally, we examined microstate sequences by assessing the probabilities of transitions from one microstate to others. To this end, we computed each participant’s transition count matrix among all microstates and divided it by the total number of transitions.

#### Statistical analysis

Statistical analyses were performed using SPSS version 27.0 (IBM Corp., Armonk, NY, United States). All statistical tests were two-tailed with a significance level set at *p* < 0.05. Sex was not included as a covariate because of the severe imbalance in sex distribution; therefore, sex-related effects could not be reliably evaluated in the present sample. Because only five participants met the obesity criterion, the obese subgroup was excluded from subsequent BMI subgroup comparisons, BV subgroup comparisons, correlation analyses, and mediation analyses to avoid unstable estimates. Therefore, all inferential statistical analyses were conducted in the remaining 118 participants.

Prior to inferential analyses, data were screened for normality and homogeneity of variance. The Shapiro–Wilk test was used to assess the normality of distributions, and Levene’s test was applied to evaluate the assumption of homogeneity of variances. Variables that met these assumptions were analyzed using parametric methods, whereas nonparametric tests were used when assumptions were violated. In addition, to improve comparability across variables and reduce potential scale effects, relevant continuous variables were standardized (z-scores) before correlation and mediation analyses.

One-way analysis of variance (ANOVA) was conducted to examine differences in temporal microstate parameters (coverage, mean duration, global explained variance (GEV), global field power [GFP], and occurrence) across the four microstate classes. To account for multiple comparisons across microstate parameters, *p*-values from the ANOVA models were corrected using the false discovery rate (FDR) method based on the Benjamini–Hochberg procedure. When a significant main effect remained after FDR correction, Tukey’s honestly significant difference (HSD) test was performed for post-hoc pairwise comparisons.

For subgroup analyses, participants were stratified according to body mass index (BMI) and blood viscosity (BV) levels. Group differences in demographic and clinical variables were examined using nonparametric tests according to the distributional characteristics of the data, while categorical variables were analyzed using chi-square tests. Given that some variables did not satisfy normality or homogeneity assumptions, nonparametric tests were applied (Mann–Whitney U test for two-group comparisons and Kruskal–Wallis test for multiple-group comparisons, as appropriate).

To further evaluate the baseline comparability of the BMI- and BV-based subgroups, demographic and clinical characteristics were compared across groups before conducting subsequent subgroup analyses. For BMI-based subgroups, sex and ethnicity were examined using chi-square tests, whereas age and BV were analyzed using Kruskal–Wallis tests because of violations of normality assumption. For BV-based subgroups, sex and ethnicity were examined using chi-square tests, and age and BMI were compared using Mann–Whitney U tests. To account for multiple testing in these baseline comparability analyses, *p*-values were adjusted using the false discovery rate (FDR) method based on the Benjamini–Hochberg procedure. Corrected *p*-values were reported in the results.

Pearson correlation analysis was used to assess the relationships among BMI, BV, microstate parameters, and transition probabilities between microstates. To control for the potential inflation of Type I error due to multiple comparisons, the resulting *p*-values were corrected using the false discovery rate (FDR) method based on the Benjamini–Hochberg procedure.

Furthermore, mediation analysis was conducted using the PROCESS macro for SPSS (Model 4) to examine the mediating role of BV in the relationship between BMI and microstate parameters. A bootstrap resampling procedure with 5,000 iterations was employed to estimate indirect effects, and bias-corrected 95% confidence intervals (CIs) were calculated. A mediation effect was considered statistically significant if the 95% CI did not include zero.

## Results

### General information

The analysis was conducted on three groups: underweight (*n* = 20), normal weight (*n* = 77), and overweight (*n* = 21). The demographic characteristics of the participants are summarized in Table 1. After excluding the obese subgroup, the final analytic sample included 118 participants, including 18 males and 100 females. The sample consisted of participants of Han (*n* = 61, 51.7%) and Tibetan ethnicity (*n* = 57, 48.3%). The mean age of the participants was 32.52 ± 8.54 years (range: 21–63 years). The mean body mass index (BMI) was 21.26 ± 2.77 kg/m^2^(range: 16.41–27.64), and the mean blood viscosity (BV) was 5.75 ± 1.04 (range: 3.47–9.80).

**TABLE 1:** Demographic and clinical characteristics of participants.

Variable	BMI group			BV group	
Parameter	Underweight (*n* = 20)	Normal weight (*n* = 77)	Overweight (*n* = 21)	Low BV (*n* = 57)	High BV (*n* = 61)
Sex					
Male	2	10	6	3	15
Female	18	67	15	54	46
Ethnicity					
Han	9	38	14	12	49
Tibetan	11	39	7	45	12
Age (mean ± SD)	30.200 ± 4.561	31.714 ± 7.614	37.667 ± 12.290	29.614 ± 5.876	35.230 ± 9.723
BMI (mean ± SD)	17.602 ± 0.673	21.005 ± 1.554	25.685 ± 1.017	20.779 ± 2.615	21.711 ± 2.852
BV (mean ± SD)	5.558 ± 0.982	5.691 ± 0.990	6.119 ± 1.223	4.973 ± 0.612	6.466 ± 0.816

Data are presented as mean ± SD or n. BV, blood viscosity; BMI, body mass index.

To evaluate the comparability of demographic characteristics across groups, statistical analyses were conducted for sex, ethnicity, age, BMI, and blood viscosity. For BMI-based subgroups, chi-square tests showed no significant differences in sex distribution (χ^2^ = 3.614, *FDR-corrected p* = 0.219) or ethnicity (χ^2^ = 2.414, *FDR-corrected p* = 0.299). In addition, nonparametric analyses indicated no significant differences among BMI groups in age (*H* = 3.391, *FDR-corrected p* = 0.459) or blood viscosity (*H* = 2.376, *FDR-corrected p* = 0.667). These findings indicate that the BMI-based subgroups were comparable with respect to the examined demographic and clinical characteristics.

For blood viscosity (BV)-based subgroups, chi-square tests revealed significant differences in sex distribution (χ^2^ = 8.514, *FDR-corrected p* = 0.007) and ethnicity (χ^2^ = 41.460, *FDR-corrected p* < 0.001). Further Mann–Whitney U tests showed a significant difference in age between BV groups (*Z* = 3.582, *FDR-corrected p* = 0.006), whereas BMI did not differ significantly between groups (*Z* = 1.810, *FDR-corrected p* = 0.332). These results suggest that the BV-based subgroups were not fully matched in demographic characteristics. Overall, the BMI-based subgroups were demographically comparable, whereas the BV-based subgroups showed differences in sex, ethnicity, and age. These differences were reported to provide a more complete description of the sample characteristics and are further acknowledged as a limitation of the subgroup analyses.

**FIGURE 1:**
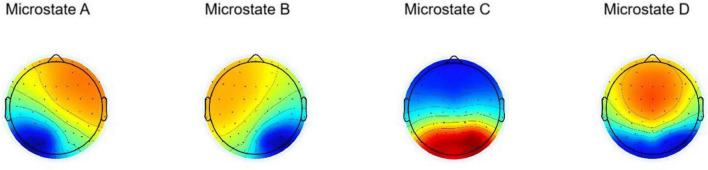
The four EEG microstate topographies identified in the present study. The microstates were derived using a polarity-invariant clustering algorithm.

The meta-criterion for determining the optimal number of microstates showed four microstates, labeled A–D (as shown in Figure 1). The topographic distribution of microstates A–D was consistent with most previous studies (Michel and Koenig, 2018). Microstate A exhibited a left–right orientation, microstate B exhibited a left–right orientation, microstate C exhibited an anterior–posterior orientation, and microstate D exhibited a maximum in the fronto-central area. Descriptive statistics for the microstate data are shown in Table 2.

**TABLE 2:** Descriptive statistics of temporal parameters for the four microstates.

Parameter	Statistic	Microstate A	Microstate B	Microstate C	Microstate D
Coverage					
	Mean ± SD	0.245 ± 0.065	0.218 ± 0.062	0.291 ± 0.082	0.247 ± 0.089
	Range	0.028–0.401	0.039–0.454	0.047–0.545	0.071–0.643
Duration					
	Mean ± SD	85.539 ± 11.428	81.605 ± 10.451	92.75 ± 17.125	88.241 ± 24.981
	Range	64.575–128.675	58.211–116.038	56.8–158.145	62.389–245.728
GEV					
	Mean ± SD	0.111 ± 0.046	0.097 ± 0.045	0.227 ± 0.093	0.16 ± 0.084
	Range	0.006–0.252	0.005–0.297	0.019–0.538	0.031–0.52
GFP					
	Mean ± SD	4.327 ± 1.51	4.294 ± 1.471	4.999 ± 1.713	4.774 ± 1.716
	Range	2.349–12.125	2.189–12.562	2.56–11.275	2.447–13.672
Occurrence					
	Mean ± SD	2.84 ± 0.607	2.645 ± 0.567	3.095 ± 0.525	2.761 ± 0.468
	Range	0.439–3.859	0.668–3.915	0.833–3.949	1.131–3.722

### Mean differences of temporal parameters among microstates

**FIGURE 2:**
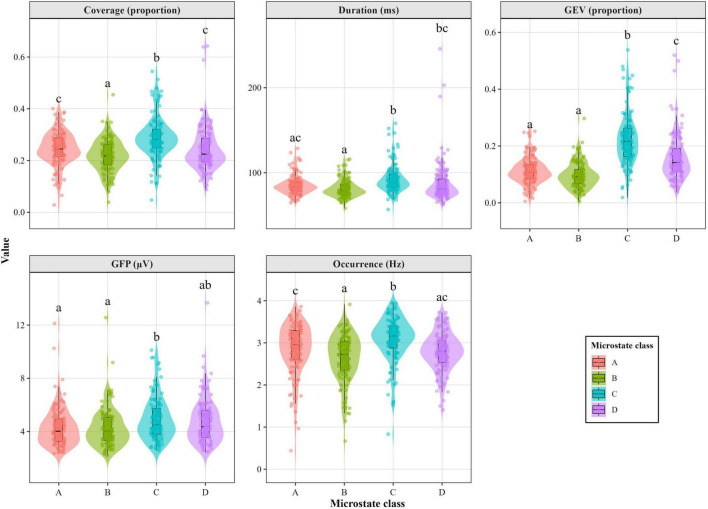
Violin plots of one-way ANOVA results for the mean coverage (proportion), duration (ms), global explained variance (GEV, proportion), global field power (GFP, μV), and occurrence (Hz) across the four microstates. Letters above each microstate (a, b, c, d) denote Tukey *post-hoc* groupings: the same letter indicates no significant difference, while different letters indicate a significant difference.

One-way analysis of variance (ANOVA) revealed significant differences in temporal microstate parameters across the four microstate classes (A–D) (as shown in Figure 2). All *p*-values reported in this section were adjusted for multiple comparisons using the false discovery rate (FDR) correction. For coverage, a significant main effect of microstate class was observed, *F*_(3, 468)_ = 18.01, *p* < 0.001. *Post-hoc* Tukey HSD tests showed that coverage was significantly higher in microstate C compared to A and B (*ps* < 0.001), and significantly higher in D compared to B (*p* = 0.024). In addition, coverage in B was significantly lower than in A (*p* = 0.036). No significant difference was found between A and D. For mean duration, the main effect of microstate class was significant, *F*_(3,__468)_ = 9.33, *p* < 0.001. *Post-hoc* comparisons indicated that microstate C had significantly longer duration than A (*p* = 0.005) and B (*p* < 0.001). Microstate D also showed longer duration than B (*p* = 0.017). No other pairwise comparisons were significant. For global explained variance (GEV), a robust main effect was found, *F*_(3__,468)_ = 79.79, *p* < 0.001. Tukey HSD results demonstrated that microstate C exhibited significantly higher GEV than all other states (A, B, and D; all *ps* < 0.001). Microstate D also showed significantly higher GEV than A and B (both *ps* < 0.001), while no significant difference was observed between A and B. For global field power (GFP), the main effect of microstate class was significant, *F*_(3,__468)_ = 5.11, *p* = 0.002. *Post-hoc* analyses revealed that GFP in microstate C was significantly higher than in A (*p* = 0.012) and B (*p* = 0.008). No other comparisons reached statistical significance. For occurrence, a significant main effect was observed, *F*_(3,__468)_ = 12.77, *p* < 0.001. Tukey HSD tests showed that occurrence was significantly higher in microstate C compared to A and B (both *ps* < 0.01). Additionally, occurrence in B was significantly lower than in A (*p* = 0.028). Microstate D did not significantly differ from A or B, but was significantly lower than C (*p* < 0.001).

### Nonparametric tests and correlation analysis

After standardizing all data using SPSS 27.0, nonparametric tests and Pearson correlation analyses were performed in the same 118 participants after excluding the obese subgroup. For both BMI-based subgroup comparisons and BV-based subgroup comparisons, no significant differences were found in any microstate parameters or transition probabilities. The detailed results are presented in Supplementary Tables 1, 2.

**FIGURE 3:**
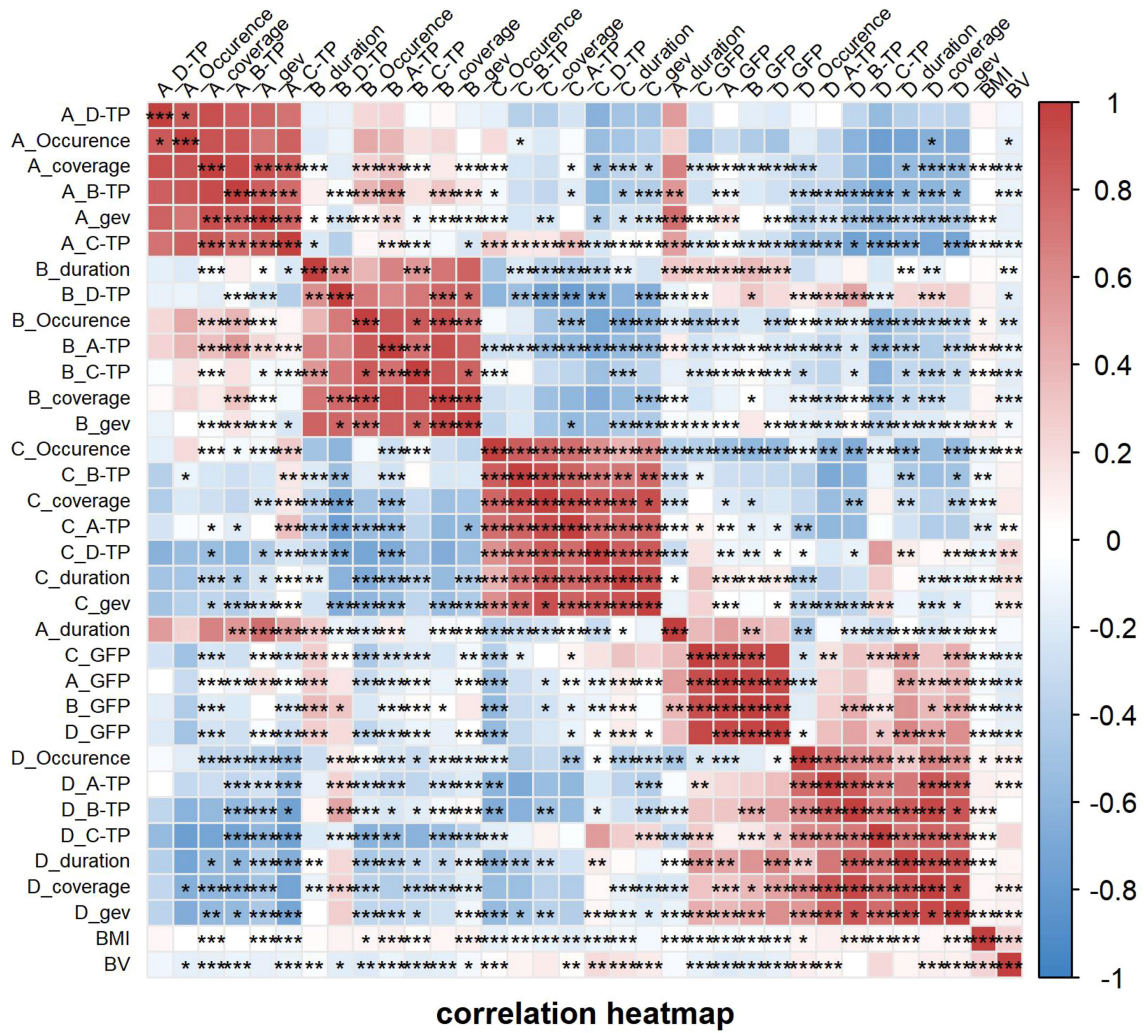
Pearson correlation matrix among BMI, estimated BV, EEG microstate parameters, and transition probabilities. Asterisks indicate correlations that remained significant after FDR correction.

*p*-values were adjusted using the Benjamini–Hochberg FDR procedure. Pearson correlation analysis showed the correlations among BMI, BV, and the coverage, duration, GEV, GFP, occurrence probability of microstates A, B, C, and D, as well as transition probabilities among microstates, as presented in Figure 3. Among them, the correlation between BMI and BV was 0.25 (corrected *p* < 0.05), and the correlation between BV and the occurrence probability of microstate B was −0.19 (corrected *p* < 0.05).

### Mediating effect of blood viscosity between BMI and the occurrence probability of microstate B

**FIGURE 4:**
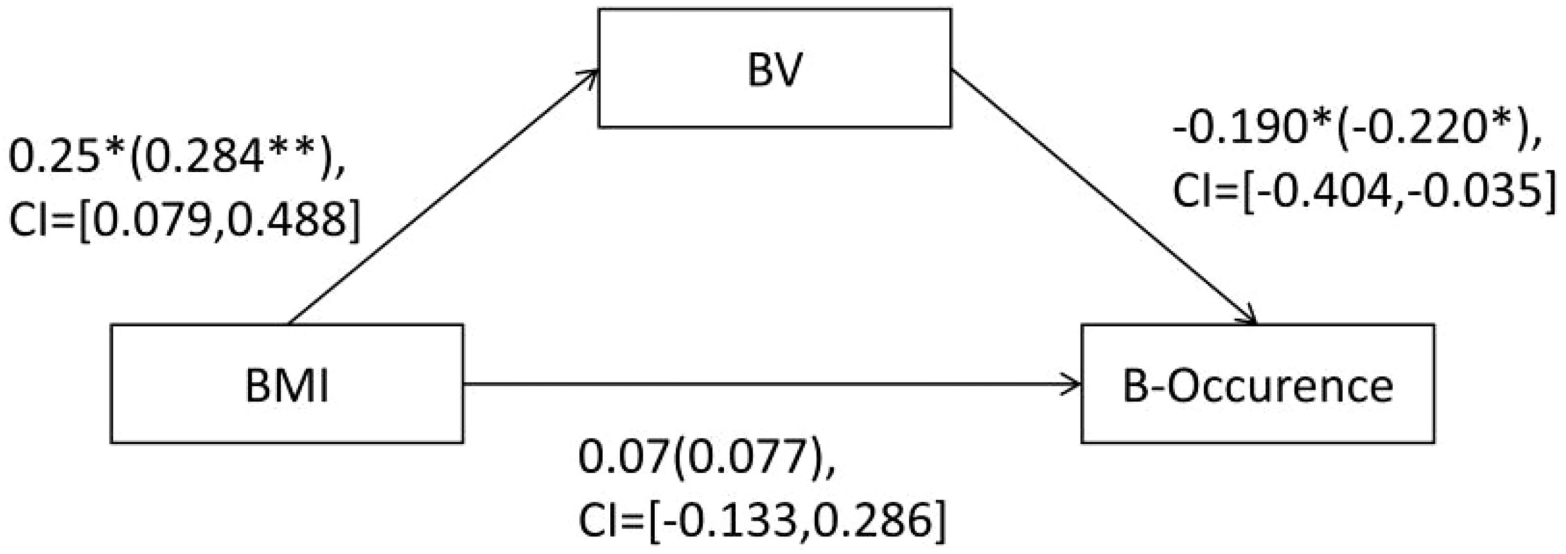
Mediation model testing the indirect statistical pathway from BMI to microstate B occurrence through estimated BV.

Bootstrap mediation analysis showed a significant indirect effect of BMI on microstate B occurrence through estimated blood viscosity. The detailed results are presented in Figure 4. Specifically, BMI significantly predicted estimated BV, *a* = 0.284, *SE* = 0.103, *t* = 2.747, *p* = 0.007, 95% CI (0.079, 0.488). Estimated BV, in turn, significantly predicted microstate B occurrence after controlling for BMI, *b* = −0.220, *SE* = 0.093, *t* = −2.353, *p* = 0.020, 95% CI [−0.404, −0.035]. The indirect effect was significant, *a × b* = −0.062, *SE* = 0.035, 95% CI [−0.142, −0.005], indicating that higher BMI was associated with higher estimated BV, which was further associated with lower microstate B occurrence. The direct effect of BMI on microstate B occurrence was not significant after including estimated BV in the model, *c’* = 0.139, *SE* = 0.107, *t* = 1.298, *p* = 0.197, 95% CI (-0.073, 0.351). The total effect of BMI on microstate B occurrence was also not significant, *c* = 0.077, *SE* = 0.106, *t* = 0.726, *p* = 0.469, 95% CI (-0.133, 0.286). The detailed results are presented in Table 3. These results suggest an indirect statistical association between BMI and microstate B occurrence through estimated BV, although the absence of a significant total effect indicates that this mediation pattern should be interpreted cautiously.

**TABLE 3:** Mediation analysis of BMI, blood viscosity, and microstate B occurrence (*n* = 118).

Predictor	*B*	SE	*t*	*p*	β
Model 1: Z (B-occurrence) ∼ Z(BMI)					
Constant	0.016	0.093	0.174	0.863	–
Z(BMI)	0.077	0.106	0.726	0.469	0.067
*R* ^2^	0.005				
Adjusted *R*^2^	−0.004				
*F*	*F*(1, 116) = 0.527				
Model 2: Z(BV) ∼ Z(BMI)					
Constant	0.027	0.09	0.303	0.762	–
Z(BMI)	0.284	0.103	2.747	0.007	0.247
*R* ^2^	0.061				
Adjusted *R*^2^	0.053				
*F*	*F*(1, 116) = 7.547				
Model 3: Z(B-occurrence) ∼ Z(BMI) + Z(BV)					
Constant	0.022	0.091	0.243	0.808	–
Z(BMI)	0.139	0.107	1.298	0.197	0.122
Z(BV)	−0.22	0.093	−2.353	0.02	−0.221
*R* ^2^	0.05				
Adjusted *R*^2^	0.034				
*F*	*F*(2, 115) = 3.041				

B, unstandardized regression coefficient; SE, standard error; β, standardized regression coefficient; BMI, body mass index; BV, blood viscosity.

## Discussion

In this study, we examined the associations among body mass index (BMI), estimated blood viscosity (BV), and resting-state EEG microstate dynamics in a high-altitude population. The results did not support a significant direct association between BMI and microstate B occurrence. Instead, they suggested a modest indirect statistical pathway: higher BMI was associated with higher estimated BV, and higher estimated BV was associated with lower occurrence of microstate B. These findings provide preliminary evidence that hemorheological status may partly link body composition to spontaneous brain-state dynamics under chronic high-altitude hypoxic exposure.

First, the topographic distributions of microstates A–D were consistent with most previous studies, and among them, microstate C showed the highest values across all parameters, which was not entirely consistent with previous studies (Bagdasarov et al., 2022), suggesting that microstate C may serve as the dominant pattern of neural activity in high-altitude environments. Our findings were consistent with previous research suggesting that blood viscosity tends to increase under high-altitude hypoxic conditions (Wan et al., 2025). The increase in blood viscosity may lead to changes in cerebral microcirculation, thereby affecting brain function.

However, in the present study, no significant differences in microstate parameters or transition probabilities were observed across BV subgroups. This suggested that the influence of blood viscosity on microstate dynamics may not be directly reflected in group comparisons, but rather operates through more subtle or continuous relationships. Therefore, the role of BV in shaping neural activity patterns may be better captured through correlation and mediation analyses rather than categorical group comparisons.

The present study also observed a positive association between BMI and estimated blood viscosity, suggesting that individuals with higher BMI may be more likely to exhibit elevated hemorheological burden under high-altitude conditions. This may be related to obesity-related inflammatory states and metabolic abnormalities (Ellulu et al., 2017). The study also found that such physiological changes may be related to individuals’ adaptation ability in high-altitude environments, and overweight individuals and those with elevated BMI may face greater physiological challenges.

Although several regression coefficients reached statistical significance, the overall R^2^ values of the models were relatively low, ranging from 0.005 to 0.061. This indicates that BMI and estimated blood viscosity explained only a small proportion of the variance in microstate B occurrence. Therefore, these variables should be interpreted as contributing factors rather than dominant determinants of microstate B dynamics. Given the multifactorial nature of electrophysiological microstate activity, additional biological, physiological, and psychosocial factors may also play important roles.

In particular, several potentially important covariates were not included in the present analyses. Blood pressure, oxygen saturation, and cerebral blood flow indices were unavailable, although these factors may influence both hemorheological status and EEG microstate dynamics under high-altitude hypoxic conditions. Education level was also not available for all participants, which limited our ability to evaluate its potential influence on resting-state brain activity. Therefore, omitted variable bias cannot be ruled out, and the observed associations among BMI, estimated blood viscosity, and microstate B occurrence should be interpreted cautiously. Future studies incorporating a broader range of physiological and demographic covariates are needed to better clarify the mechanisms underlying BMI-related microstate alterations.

The mediation model suggested an indirect statistical association between BMI, estimated blood viscosity, and the occurrence probability of microstate B. This finding indicates that the association between BMI and spontaneous brain activity may be partly related to hemorheological changes. Microstate B has been associated with externally oriented sensory-cognitive processes, and simultaneous EEG-fMRI and high-density EEG source-imaging studies have suggested that occipital regions, including the visual cortex, are important generators of microstate B (Baradits et al., 2020; Michel and Koenig, 2018). One possible explanation is that increased blood viscosity may affect cerebral blood-flow supply or oxygen-delivery efficiency, thereby influencing the temporal expression of microstate B. However, this interpretation remains speculative because cerebral perfusion and oxygenation measures were not directly assessed in the present study.

These findings suggest that hemorheological status may be a potential factor for future observational and interventional studies in high-altitude environments. Although BV may be more modifiable than BMI in some physiological or clinical contexts, the present cross-sectional data do not support direct clinical recommendations. Future longitudinal or interventional studies are required to determine whether regulating hemorheological status can benefit brain function in high-altitude populations.

### Limitations and future research directions

This study has several limitations that should be acknowledged. First, as the study was conducted in a specific high-altitude environment, the findings may not be fully generalizable to populations living at other altitudes or under different environmental conditions. Second, the present study did not include a control group under normoxic or low-altitude conditions. As a result, it remains unclear to what extent the observed relationships among BMI, blood viscosity, and EEG microstate dynamics are specific to the hypoxic environment of high altitude, or whether similar patterns would also be present under normal conditions. Future studies incorporating low-altitude control groups or within-subject comparisons across different altitudes are needed to clarify the environmental specificity of the observed effects. Third, the cross-sectional design of this study precludes any causal inference regarding the relationships among BMI, blood viscosity, and EEG microstate parameters. Future studies employing longitudinal designs are needed to further validate these associations and clarify their causal directions. Fourth, the sample exhibited a noticeable sex imbalance, with substantially fewer male participants than female participants. Given that sex differences may influence body composition, hemorheological properties, and brain functional activity, the potential impact of sex on the present findings cannot be ruled out. Fifth, blood viscosity was estimated using a previously published high-altitude prediction model rather than directly measured. Although this model was developed in a high-altitude population and showed acceptable predictive performance, estimated BV cannot fully replace direct rheological measurement. Future studies should directly measure blood viscosity to validate the present findings. Finally, although several unmeasured covariates have been discussed above, the absence of direct blood-pressure, oxygen-saturation, cerebral blood-flow, and complete education-level data remains an important limitation and may have introduced omitted variable bias. Future studies should recruit larger and more balanced samples, incorporate low-altitude comparison groups, directly measure relevant physiological variables, and examine additional potential mediating factors to improve model validity and generalizability.

## Conclusion

This study found that BMI was positively associated with estimated BV, whereas estimated BV was negatively associated with the occurrence of EEG microstate B in high-altitude residents. The mediation analysis suggested an indirect statistical association linking BMI to microstate B occurrence through estimated BV. In addition, microstate C showed relatively higher temporal parameters, suggesting a potentially dominant resting-state pattern under high-altitude conditions. These findings provide preliminary evidence for a BMI–BV–EEG microstate pathway in high-altitude residents. However, because of the cross-sectional design, low explained variance, estimated rather than directly measured BV, absence of several key covariates, and lack of a low-altitude control group, the findings should be interpreted cautiously.

## Data Availability

The data analyzed in this study is subject to the following licenses/restrictions. Requests to access these datasets should be directed to liuzianziannliu@163.com.
