# Supplementary material for: Body mass index affects EEG microstate dynamics through blood viscosity in high-altitude environments
Source: Front Neurosci. 2026 Jun 19;20:1829857. doi: 10.3389/fnins.2026.1829857 (PMC13330577; doi:10.3389/fnins.2026.1829857)
Supplement: Supplementary file 1 [file Supplementary_file_1.docx]

**Supplementary Material**

Supplementary Table 1 presents the nonparametric comparison results of EEG microstate parameters and transition probabilities across BMI-based subgroups, including the underweight, normal-weight, and overweight groups. Supplementary Table 2 presents the corresponding nonparametric comparison results between the low-BV and high-BV subgroups. All analyses were conducted in the final analytic sample of 118 participants after excluding the obese subgroup, and p-values were adjusted using the Benjamini–Hochberg false discovery rate procedure. No significant subgroup differences were observed in any microstate parameters or transition probabilities after correction.

Supplementary Table 1. Nonparametric comparison results across BMI-based subgroups.

|  | BMI group (M±SD) | | | Kruskal-Wallis H | *FDR-corrected* |
| --- | --- | --- | --- | --- | --- |
|  | Underweight (*n*=20) | Normal weight (*n*=77) | Overweight (*n*=21) |  |  |
| A_coverage | 0.251 ± 0.06 | 0.242 ± 0.064 | 0.251 ± 0.081 | 0.086 | 0.992 |
| B_coverage | 0.213 ± 0.063 | 0.218 ± 0.061 | 0.222 ± 0.07 | 0.468 | 0.992 |
| C_coverage | 0.284 ± 0.07 | 0.3 ± 0.083 | 0.258 ± 0.087 | 3.935 | 0.449 |
| D_coverage | 0.252 ± 0.078 | 0.239 ± 0.091 | 0.27 ± 0.09 | 3.527 | 0.449 |
| A_duration | 88.051 ± 14.865 | 84.225 ± 10.254 | 87.348 ± 11.718 | 1.372 | 0.938 |
| B_duration | 82.132 ± 12.052 | 80.93 ± 10.041 | 82.763 ± 10.786 | 0.726 | 0.992 |
| C_duration | 90.878 ± 13.35 | 94.281 ± 17.569 | 87.53 ± 18.809 | 5.281 | 0.449 |
| D_duration | 87.345 ± 16.097 | 87.292 ± 26.035 | 91.031 ± 26.534 | 1.11 | 0.976 |
| A_gev | 0.117 ± 0.055 | 0.109 ± 0.042 | 0.119 ± 0.055 | 0.522 | 0.992 |
| B_gev | 0.092 ± 0.047 | 0.097 ± 0.042 | 0.103 ± 0.055 | 0.305 | 0.992 |
| C_gev | 0.22 ± 0.085 | 0.234 ± 0.094 | 0.202 ± 0.097 | 2.655 | 0.644 |
| D_gev | 0.165 ± 0.077 | 0.154 ± 0.087 | 0.181 ± 0.08 | 4.321 | 0.449 |
| A_GFP | 4.263 ± 1.361 | 4.344 ± 1.588 | 4.227 ± 1.197 | 0.053 | 0.992 |
| B_GFP | 4.149 ± 1.263 | 4.334 ± 1.574 | 4.199 ± 1.177 | 0.282 | 0.992 |
| C_GFP | 4.904 ± 1.593 | 5.027 ± 1.762 | 4.84 ± 1.495 | 0.134 | 0.992 |
| D_GFP | 4.674 ± 1.439 | 4.813 ± 1.833 | 4.655 ± 1.387 | 0.017 | 0.992 |
| A_Occurrence | 2.849 ± 0.485 | 2.851 ± 0.629 | 2.832 ± 0.688 | 0.146 | 0.992 |
| B_Occurrence | 2.572 ± 0.556 | 2.671 ± 0.583 | 2.645 ± 0.529 | 1.292 | 0.938 |
| C_Occurrence | 3.104 ± 0.458 | 3.148 ± 0.504 | 2.893 ± 0.613 | 3.526 | 0.449 |
| D_Occurrence | 2.838 ± 0.528 | 2.704 ± 0.448 | 2.947 ± 0.331 | 5.73 | 0.449 |
| A_B-TP | 0.344 ± 0.063 | 0.335 ± 0.076 | 0.336 ± 0.088 | 0.058 | 0.992 |
| A_C-TP | 0.341 ± 0.074 | 0.346 ± 0.083 | 0.324 ± 0.088 | 0.961 | 0.992 |
| A_D-TP | 0.318 ± 0.067 | 0.309 ± 0.071 | 0.333 ± 0.083 | 1.596 | 0.9 |
| B_A-TP | 0.305 ± 0.076 | 0.308 ± 0.076 | 0.318 ± 0.078 | 0.015 | 0.992 |
| B_C-TP | 0.291 ± 0.076 | 0.31 ± 0.07 | 0.294 ± 0.059 | 3.639 | 0.449 |
| B_D-TP | 0.283 ± 0.065 | 0.292 ± 0.077 | 0.302 ± 0.093 | 0.137 | 0.992 |
| C_A-TP | 0.379 ± 0.074 | 0.392 ± 0.087 | 0.338 ± 0.094 | 3.885 | 0.449 |
| C_B-TP | 0.343 ± 0.074 | 0.365 ± 0.08 | 0.325 ± 0.074 | 4.75 | 0.449 |
| C_D-TP | 0.4 ± 0.08 | 0.399 ± 0.104 | 0.365 ± 0.099 | 1.876 | 0.832 |
| D_A-TP | 0.317 ± 0.071 | 0.3 ± 0.079 | 0.344 ± 0.06 | 8.807 | 0.208 |
| D_B-TP | 0.314 ± 0.079 | 0.3 ± 0.093 | 0.339 ± 0.1 | 3.793 | 0.449 |
| D_C-TP | 0.368 ± 0.099 | 0.344 ± 0.104 | 0.383 ± 0.106 | 4.956 | 0.449 |
| p-values were adjusted using the Benjamini–Hochberg FDR procedure. | | | | | |

Supplementary Table 2. Nonparametric comparison results across BV-based subgroups

|  | BV group (M±SD) | | Mann-Whitney Z | *FDR-corrected* |
| --- | --- | --- | --- | --- |
|  | High Blood Viscosity(*n*=61) | Low Blood Viscosity(*n*=57) |  |  |
| A_coverage | 0.238 ± 0.063 | 0.252 ± 0.069 | -1.661 | 0.345 |
| B_coverage | 0.211 ± 0.062 | 0.226 ± 0.062 | -1.161 | 0.395 |
| C_coverage | 0.3 ± 0.085 | 0.279 ± 0.079 | 1.047 | 0.395 |
| D_coverage | 0.25 ± 0.067 | 0.243 ± 0.107 | 1.694 | 0.345 |
| A_duration | 84.499 ± 10.842 | 86.425 ± 12.026 | -0.934 | 0.41 |
| B_duration | 80.507 ± 9.725 | 82.48 ± 11.202 | -0.837 | 0.441 |
| C_duration | 94.499 ± 18.174 | 90.367 ± 16.044 | 1.187 | 0.395 |
| D_duration | 87.558 ± 15.059 | 88.403 ± 31.941 | 1.683 | 0.345 |
| A_gev | 0.106 ± 0.047 | 0.118 ± 0.046 | -1.548 | 0.345 |
| B_gev | 0.094 ± 0.045 | 0.101 ± 0.045 | -0.859 | 0.441 |
| C_gev | 0.237 ± 0.098 | 0.214 ± 0.086 | 1.134 | 0.395 |
| D_gev | 0.165 ± 0.068 | 0.156 ± 0.098 | 1.704 | 0.345 |
| A_GFP | 4.044 ± 1.111 | 4.593 ± 1.757 | -1.661 | 0.345 |
| B_GFP | 4.044 ± 1.111 | 4.529 ± 1.722 | -1.478 | 0.345 |
| C_GFP | 4.748 ± 1.373 | 5.214 ± 1.937 | -0.988 | 0.395 |
| D_GFP | 4.543 ± 1.348 | 4.995 ± 1.977 | -0.983 | 0.395 |
| A_Occurrence | 2.797 ± 0.57 | 2.901 ± 0.658 | -1.306 | 0.395 |
| B_Occurrence | 2.587 ± 0.587 | 2.716 ± 0.54 | -1.074 | 0.395 |
| C_Occurrence | 3.14 ± 0.45 | 3.047 ± 0.589 | 0.471 | 0.657 |
| D_Occurrence | 2.824 ± 0.428 | 2.712 ± 0.471 | 1.026 | 0.395 |
| A_B-TP | 0.33 ± 0.07 | 0.344 ± 0.081 | -1.556 | 0.345 |
| A_C-TP | 0.335 ± 0.079 | 0.347 ± 0.086 | -1.505 | 0.345 |
| A_D-TP | 0.309 ± 0.07 | 0.321 ± 0.076 | -1.349 | 0.395 |
| B_A-TP | 0.301 ± 0.072 | 0.317 ± 0.079 | -1.161 | 0.395 |
| B_C-TP | 0.297 ± 0.07 | 0.312 ± 0.068 | -1.112 | 0.395 |
| B_D-TP | 0.283 ± 0.075 | 0.303 ± 0.081 | -0.751 | 0.481 |
| C_A-TP | 0.384 ± 0.086 | 0.377 ± 0.09 | 0.013 | 0.989 |
| C_B-TP | 0.361 ± 0.074 | 0.346 ± 0.083 | 1.01 | 0.395 |
| C_D-TP | 0.408 ± 0.102 | 0.376 ± 0.094 | 1.478 | 0.345 |
| D_A-TP | 0.315 ± 0.063 | 0.306 ± 0.088 | 1.468 | 0.345 |
| D_B-TP | 0.309 ± 0.068 | 0.309 ± 0.114 | 1.279 | 0.395 |
| D_C-TP | 0.368 ± 0.1 | 0.341 ± 0.106 | 1.78 | 0.345 |

p-values were adjusted using the Benjamini–Hochberg FDR procedure.
